# A Proposal of Secure and Automated Over-the-Air Firmware Update Mechanism for IoT Devices Using Continuous Integration and Continuous Delivery

**DOI:** 10.3390/s26051535

**Published:** 2026-02-28

**Authors:** Noprianto, Nobuo Funabiki, Htoo Htoo Sandi Kyaw, Komang Candra Brata, I Nyoman Darma Kotama

**Affiliations:** Department of Information and Communication Systems, Okayama University, Okayama 700-8530, Japan; noprianto@s.okayama-u.ac.jp (N.); htoohtoo@okayama-u.ac.jp (H.H.S.K.); k.candra.brata@ub.ac.id (K.C.B.); p9363bg2@s.okayama-u.ac.jp (I.N.D.K.)

**Keywords:** *Internet of Things (IoT)*, *over-the-air (OTA)* firmware update, security, *continuous integration (CI)*, *continuous delivery (CD)*

## Abstract

The *Internet of Things (IoT)* technology has grown rapidly over the past decade, resulting in deployments of thousands of *IoT* devices around the world. Then, managing firmware updates for these numerous devices poses significant challenges. Firmware updates face issues such as *version rollback*, *modified firmware files*, and *potential man-in-the-middle (MITM) attacks*, highlighting the need for a *secure over-the-air (OTA) firmware update* mechanism. In this paper, we propose an *automated OTA firmware update mechanism*, integrated with *continuous integration (CI)* and *continuous delivery (CD)* to ensure trusted sources for firmware origins. It offers security, error handling during firmware updates, and monitoring of the update process. For evaluations, we implemented the proposal with the *SEMAR IoT* application server that has been implemented in our previous studies. Then, we verified the integrity and authentication, measured the performance and resource utilization, and performed benchmarking tests to assess the efficiency. The results demonstrate that the proposal is sufficiently reliable and efficient.

## 1. Introduction

The *Internet of Things (IoT)* has experienced rapid growth over the past decade. It has been deployed in diverse domains including healthcare, industrial automation, automotive systems, and military applications [1,2,3,4]. The *IoT* scale reached approximately 30 billion connected devices in 2024 around the world [5], creating unprecedented challenges in large-scale device management. In addition to large-scale device management challenges, edge computing paradigms such as *Mobile Edge Computing (MEC)* have been increasingly adopted to support low-latency, secure, and resource-efficient *IoT* operations by enabling computation offloading and distributed processing near end devices [6]. This trend highlights the growing importance of distributed architectures in modern *IoT* ecosystems. When hundreds or thousands of *IoT* devices are deployed in the field, firmware update management becomes a critical operational challenge. *Over-the-air (OTA)* firmware updates via wireless networks have emerged as the preferred method, replacing conventional approaches that relied on physical connectivity such as USB or UART interfaces. Regular firmware updates are essential for addressing software bugs, enhancing device performance, and patching security vulnerabilities in *IoT* devices.

In the context of firmware updates, security is a paramount challenge, particularly for *IoT* devices employed in critical applications such as military or healthcare systems that are intolerant to failures. One of the most serious security threats is *MITM* attacks, where adversaries can intercept and modify firmware files during transmission [7,8,9,10,11]. These attacks can result in firmware modifications with malicious codes, firmware version downgrades (rollback attacks), or code injections for specific malicious purposes. Wireless communication channels are highly vulnerable to *MITM* attacks due to limited physical control, necessitating robust security mechanisms to ensure firmware integrity and authenticity.

Recent large-scale empirical studies on consumer *IoT* devices further reveal that authentication weaknesses remain widespread in practice, even when TLS is implemented [12]. In particular, improper certificate validation, insufficient trust anchor verification, and replay vulnerabilities have been identified as common failure points in deployed devices. These findings suggest that the mere presence of cryptographic protocols does not guarantee secure firmware distribution. Therefore, *OTA* update mechanisms must enforce strict certificate validation, authenticated firmware origin verification, and version monotonicity checks to address concrete weaknesses observed in real-world *IoT* deployments.

Various research efforts have been conducted to secure the *OTA* update process for *IoT* devices through cryptographic approaches and distribution optimization. From the data security perspective, several studies have employed dual-XOR operations with *DEFLATE* compression to improve efficiency [13], *Elliptic Curve Cryptography (ECC)* and *SHA-512* algorithms to secure communications [14,15], and hybrid *ECC-AES* encryption for vehicular systems [16]. From the infrastructure perspective, version control systems have been implemented on gateways to ensure distribution of the latest firmware, with *LUKS* encryption and *Trusted Platform Module (TPM)* for access control [5]. Although these research efforts have optimized *OTA* distribution and security, most have not fully integrated with the development process, such as automated firmware building and deployment through *CI* and *CD* pipelines. This gap demonstrates the need for an approach that integrates *OTA* security with the development process in an end-to-end manner.

In this paper, we propose a fully automated *over-the-air (OTA)* firmware update mechanism that is integrated with the development process. Unlike previous research focusing on the distribution stage, the proposed approach integrates the entire pipeline from the moment when developers commit code fixes through the version control system until the firmware is deployed to the *IoT* device. The firmware build process is performed automatically on a cloud-based version control server, including secure management for storing secrets and keys used in the firmware digital signature process. This *CI* and *CD* approach ensures that distributed firmware truly originates from trusted sources and has undergone rigorous verification processes. In the implementation, the proposed mechanism is integrated with the *SEMAR IoT* application server that we have developed in previous studies. The implemented system offers comprehensive security features, error handling during the update process, and real-time monitoring of firmware update status on *IoT* devices.

To validate the proposed mechanism, this paper conducted comprehensive evaluations, including security testing for integrity and authentication verification, performance metrics and resource utilization measurements, and benchmarking to compare performance with existing methods. The results demonstrate that the proposed *OTA* firmware update mechanism achieves a high level of security with minimal overhead, while exhibiting superior success rates and efficiency compared to conventional approaches. The main contributions of this paper are summarized as follows:

- This paper proposes an end-to-end secure *OTA* firmware update mechanism that tightly integrates the development process with automated *CI/CD* pipelines, ensuring firmware authenticity and integrity from source code commit to device deployment while addressing known real-world authentication weaknesses in *IoT* firmware distribution.
- This paper implements the proposed mechanism on resource-constrained ESP32 devices and integrates it with the *SEMAR IoT* application server for firmware package management, including upload, storage, and controlled distribution.
- This paper conducts extensive experimental evaluations, including security verification, execution time analysis, and energy and power consumption measurements, to assess the performance and efficiency of different digital signature algorithms.

The remainder of this paper is structured as follows. Section 2 reviews related work on secure firmware update mechanisms for *IoT* devices. Section 3 presents the automated *OTA* firmware update mechanism with *CI/CD* integration. Section 4 describes the experimental setup and evaluation results. Finally, Section 5 concludes the paper and discusses future work.

## 2. Related Work

In this section, we introduce an overview of key findings from previous studies to support the discussions in this paper.

### 2.1. Cryptographic Approaches for Firmware Security

Various cryptographic approaches have been employed to secure firmware and mitigate *MITM* attacks. Several studies have explored different cryptographic techniques to ensure firmware integrity and authenticity during the update process.

In [13], Park et al. proposed a secure and lightweight *OTA* firmware update mechanism to mitigate man-in-the-middle attacks in resource-constrained *IoT* environments. The proposed approach combines dual-XOR operations with *DEFLATE* compression to reduce firmware file size and encryption overhead, while leveraging multiple transmission channels to enhance security. Experimental evaluations demonstrate improvements in latency, memory usage, and power consumption compared to conventional *OTA* methods, while maintaining robustness against brute-force attacks. However, the proposed mechanism primarily focuses on optimizing firmware transmission efficiency and lightweight encryption at the communication level, without addressing broader system-level *OTA* management or integration with the firmware development and deployment workflow.

In [14], Ben et al. proposed a low-cost and secure *OTA* firmware update framework for *IoT* devices based on the *Firmware Over-The-Air (FOTA)* technique. The framework supports broadcasting firmware updates to multiple nodes and employs cryptographic mechanisms such as *Elliptic Curve Cryptography (ECC)* and *SHA-512* to ensure secure communication. In addition, lossless data compression is applied to reduce firmware transfer time and memory usage, while enabling dynamic addition and removal of nodes within the network. Experimental results demonstrate reduced network downtime and improved continuity of *IoT* system operation. However, the proposed framework mainly concentrates on secure firmware distribution and device-level update strategies.

In [16], Cheng et al. investigated the application of a hybrid encryption scheme for securing *Firmware Over-The-Air (FOTA)* updates in vehicular systems. The proposed approach combines *Elliptic Curve Cryptography (ECC)* for key exchange with the *Advanced Encryption Standard (AES)* for encrypting firmware data, leveraging the advantages of both asymmetric and symmetric cryptography. The hybrid scheme is integrated into the transmission and reception modules of the vehicle *FOTA* system to protect firmware updates against malicious interception, data theft, and tampering during transmission. Experimental evaluations demonstrate that the proposed method enhances system security while preserving update timeliness. However, the study primarily focuses on cryptographic protection during firmware transmission.

In [17], Rozlomii et al. proposed a lightweight firmware integrity verification method for *IoT* devices with limited computational resources and power constraints. The approach employs lightweight hash functions, including *SPONGENT*, *PHOTON*, *QUARK*, and *LESAMNTA-LW*, and introduces a multi-level verification scheme based on the security criticality of firmware segments. To mitigate replay attacks, session markers are integrated into the hashing process. Experimental results on low-end microcontrollers demonstrate efficient verification with low memory and power consumption. However, the proposed method primarily focuses on firmware integrity verification and secure boot, and does not address the complete *OTA* update process or its integration with automated deployment workflows.

In [18], Schneider et al. conducted an in-depth analysis of fault attacks targeting elliptic curve-based digital signature verification schemes used in secure boot and firmware update mechanisms. The study demonstrates that implementation-level fault injections can enable forged signatures in schemes such as *ECDSA*, *EdDSA*, *ECGDSA*, and *ECSDSA*, even in hardened bootloader environments. The authors also propose effective countermeasures to strengthen signature verification against such fault attacks. However, the work mainly focuses on cryptographic vulnerability analysis and countermeasures.

Overall, existing cryptographic approaches for *OTA* firmware security primarily concentrate on transmission-level protection, lightweight encryption efficiency, or integrity verification mechanisms tailored for resource-constrained devices. While these studies significantly enhance communication security and firmware authenticity, they generally treat cryptographic protection as an isolated component of the update process. Limited attention is given to the integration of cryptographic mechanisms within a fully automated development-to-deployment workflow, including secure key management, reproducible builds, and *CI/CD-based* firmware release control. Consequently, an end-to-end framework that unifies cryptographic assurance with automated build and deployment pipelines remains insufficiently explored.

### 2.2. Over-the-Air Update Mechanisms

Several studies have investigated *OTA* firmware update mechanisms for *IoT* devices, focusing on efficient distribution strategies and reliable update protocols.

In [19], Shin et al. proposed *MQTree*, a secure *OTA* firmware update protocol for software-defined vehicles that combines the *MQTT* protocol secured with *TLS* and *Merkle* tree-based verification to ensure firmware integrity. By leveraging the lightweight messaging characteristics of *MQTT* and the tamper-detection capability of Merkle trees, the proposed protocol achieves reliable and efficient firmware updates for automotive environments. Security evaluations demonstrate resistance against attacks such as spoofing, man-in-the-middle, and duplicate updates, while performance comparisons show significantly faster processing time than conventional *HTTPS*-based *OTA* approaches. However, the proposed protocol mainly focuses on secure firmware distribution at the communication level and does not explicitly address end-to-end *OTA* automation, including firmware build, signing, and deployment workflows.

In [20], Sumi et al. proposed a firmware distribution method based on erasure codes for large-scale *IoT* networks operating on *IEEE 802.15.4g* mesh networks in Sub-1 GHz LPWAN environments. The proposed method addresses the challenge of efficiently distributing firmware to a large number of devices during network operation by reducing the overhead of unicast transmissions while avoiding the lack of delivery confirmation inherent in broadcast-based approaches. Simulation results using *ns-3* demonstrate that the method improves firmware distribution efficiency by up to 1.8 times compared to conventional schemes and achieves higher spectrum efficiency for *IEEE 802.15.4g*-OFDM physical layers. However, the work mainly focuses on optimizing firmware dissemination at the network layer and does not consider security mechanisms or integration with automated *OTA* update workflows.

In [21], Jewsakul et al. introduced *FioRa*, an energy-neutrality-aware multicast firmware distribution framework for energy-harvesting *LoRa* networks. The framework adapts the firmware dissemination process according to the predicted energy availability of sensor nodes, thereby reducing failed firmware reconstructions and unnecessary retransmissions. By employing one-hop neighbor discovery and on-demand relay mechanisms, *FioRa* significantly improves distribution performance, achieving up to 17 times shorter distribution time and 7 times lower communication overhead compared to existing approaches. Nevertheless, the proposed framework primarily targets energy-efficient firmware dissemination and does not address security aspects such as firmware authenticity verification or integration with automated *OTA* update workflows.

In [22], Nikic et al. presented an efficient implementation of differential (delta) firmware updates for *IoT* devices, with a performance analysis across *WLAN* and *LPWAN* communication technologies. The proposed approach addresses the limited data rate of *LPWAN* links by transmitting only the differences between successive firmware versions, thereby significantly reducing the amount of data transferred, bandwidth consumption, and firmware update time. Experimental evaluations demonstrate that delta-based updates are particularly effective for resource-constrained devices operating over low-throughput wireless channels. However, the study mainly focuses on optimizing firmware transfer efficiency and does not explicitly consider security mechanisms such as firmware authentication, integrity protection, or integration with automated *OTA* update pipelines.

In [23], Striegel et al. proposed *Box*, a secure firmware and key distribution system for wireless sensor networks based on an intelligent *Faraday Cage*. The system retrieves firmware images and cryptographic keys from a backend server, embeds the keys into the firmware, and deploys customized images *over-the-air* to multiple sensor nodes placed inside the Faraday Cage. Electromagnetic shielding provided by the Faraday Cage protects the firmware and key exchange against passive eavesdropping attacks. The authors demonstrated the feasibility of batch firmware deployment on commercial off-the-shelf sensor nodes, with user studies indicating that the proposed approach is faster and more user-friendly than conventional wired firmware distribution. However, the system is primarily designed for initial deployment in controlled physical environments and does not address secure remote *OTA* updates or continuous firmware lifecycle management in large-scale *IoT* deployments.

Collectively, existing *OTA* update mechanisms primarily concentrate on optimizing firmware dissemination efficiency, communication reliability, energy awareness, or bandwidth reduction through techniques such as multicast strategies, erasure coding, and differential updates. While these approaches significantly enhance distribution performance under constrained network conditions, security mechanisms are often treated as complementary components rather than integral elements of the entire firmware lifecycle. Moreover, most studies isolate the distribution phase from earlier development stages, leaving firmware build automation, secure signing processes, and controlled release management largely unaddressed. Therefore, a comprehensive *OTA* framework that simultaneously ensures secure firmware authenticity, lifecycle-aware version control, and seamless *CI/CD* integration remains insufficiently explored in the current literature.

### 2.3. CI/CD Integration in IoT Systems

The integration of *CI/CD* practices in *IoT* systems has gained attention as a means to automate firmware development and deployment processes.

In [5], Bhargava et al. proposed a version control-based gateway to optimize *OTA* firmware updates for *IoT* devices operating in environments with limited Internet connectivity. The proposed gateway leverages local version control repositories to minimize data transfer by reusing existing firmware artifacts, thereby reducing bandwidth consumption and update overhead in remote deployment scenarios. The approach follows the paradigm of “using more computing to move less data,” making it suitable for large-scale *IoT* systems deployed in connectivity-constrained environments. However, the work primarily focuses on bandwidth-efficient firmware distribution and does not explicitly address end-to-end *OTA* security mechanisms or integration with automated firmware build and deployment pipelines.

In [24], Yulianto et al. presented an automated deployment pipeline for containerized *IoT* applications using a combination of *GitLab CI* and *Jenkins*. The study implemented the pipeline following an agile development approach and evaluated its performance on backend and frontend applications deployed on a cloud platform. Experimental results showed average deployment times of 153.7 s for backend services and 174.2 s for frontend services, with pipeline success rates of 70.45% and 83.3%, respectively, demonstrating the effectiveness of integrating multiple *CI/CD* tools for automating container-based application deployments. However, the work focuses on application-level container deployment and does not address firmware-level *OTA* updates, security mechanisms, or integration with resource-constrained *IoT* devices.

In [25], Singh et al. investigated *DevOps*-based migration aspects from legacy version control systems to distributed version control systems, particularly *Git*, for microservices deployment. The study analyzed several pre-migration validation mechanisms, including project structure validation, user and author mapping between version control systems, remote server storage pre-validation, and network connectivity sensing using a *NodeMCU*-based *IoT* platform. Various DevOps tools such as *Kubernetes*, *Jenkins*, *Chef*, and *AppDynamics* were examined to support infrastructure provisioning, monitoring, and migration reliability. The results highlight that while existing DevOps tools can accelerate migration processes, they do not provide comprehensive, automated validation for ensuring full compatibility during version control system migration. However, the work primarily focuses on software repository migration and DevOps practices, and does not address secure *OTA* firmware updates, resource-constrained *IoT* devices, or automated firmware delivery pipelines.

In [26], Hu et al. proposed *GitFL*, a *Git*-inspired version control framework for managing asynchronous *Federated Learning (FL)* in *AIoT* systems. The framework adopts master–branch model management, where the global model is maintained as a master branch and local models trained by distributed devices are treated as branch models with version information. By incorporating a reinforcement learning–based device selection mechanism, GitFL effectively mitigates model staleness and balances computational load among heterogeneous and straggling devices. Experimental results demonstrate that GitFL achieves up to 2.64× training acceleration and improves inference accuracy by up to 7.88% compared to state-of-the-art synchronous and asynchronous FL methods. However, the proposed framework focuses on versioned model management for distributed learning and does not address firmware *OTA* update mechanisms, embedded device constraints, or secure firmware deployment pipelines.

Overall, existing studies on *CI/CD* integration in *IoT* systems predominantly focus on application-level deployment, container orchestration, version control optimization, or distributed model management. While these works demonstrate the benefits of DevOps practices in improving automation, scalability, and development efficiency, they rarely extend *CI/CD* principles to firmware-level update processes for resource-constrained embedded devices. In particular, the integration of automated firmware building, secure digital signing, version-aware release control, and *OTA* deployment within a unified *CI/CD* pipeline remains largely unexplored. Therefore, a secure end-to-end *OTA* framework that tightly couples firmware development workflows with automated *CI/CD* processes represents an important yet underdeveloped research direction.

### 2.4. Firmware Update Security Frameworks

Comprehensive security frameworks have been proposed to protect the entire firmware update process in *IoT* devices, encompassing end-to-end security mechanisms from firmware generation to deployment.

In [27], Shahid et al. proposed an end-to-end security framework for the *Industrial Internet of Things (IIoT)* based on a decentralized blockchain architecture. The proposed mechanism employs blockchain to provide authentication, authorization, and data integrity across highly distributed industrial devices without relying on a central authority. Smart contracts are utilized to enforce security policies with dynamic rule switching, enabling the system to respond promptly to suspicious behavior. To accommodate resource-constrained IIoT devices, the framework adopts lightweight cryptographic schemes and introduces a hybrid blockchain design that combines features of both private and public blockchains. However, the work primarily focuses on securing IIoT communications and access control, and does not address secure firmware *OTA* update processes or their integration with automated firmware build and deployment pipelines.

In [28], Tran et al. proposed a lightweight end-to-end security protocol for resource-constrained *IoT* devices based on *Challenge-Response Pair (CRP) Physically Unclonable Functions (PUFs)* and a *True Random Number Generator (TRNG)*. The protocol eliminates the need for non-volatile key storage by dynamically deriving all secret keys from hardware-based entropy sources, thereby improving resistance to physical attacks and key extraction. By replacing conventional public key authentication with *CRP-PUFs* and *hash-based message authentication codes (HMACs)*, the proposed scheme significantly reduces computational complexity while providing authentication, integrity, confidentiality, and non-repudiation. Formal verification and hardware implementation results demonstrate its feasibility and efficiency compared to traditional *ECC*-based approaches. However, the work focuses on secure device-to-device communication and key management, and does not address secure *OTA* firmware update mechanisms or their integration with automated firmware build and deployment workflows.

In [29], Younis et al. introduced *LiSB* (Lightweight Secure Boot and Attestation Scheme), a lightweight approach for validating software and firmware integrity and ensuring secure boot-up on resource-constrained embedded devices. The scheme employs *Physically Unclonable Functions (PUFs)* as a hardware *Root-of-Trust* to generate device-specific integrity digests without storing secret keys in device memory. Experimental results based on a prototype implementation demonstrate that *LiSB* outperforms *TPM*-based attestation schemes and consumes up to 25 times less power than *SHA-256*, which is commonly used in existing attestation mechanisms. However, the proposed scheme primarily focuses on secure boot and attestation at device startup, and does not address secure *OTA* firmware update processes or their integration with automated firmware build and deployment pipelines.

In [30], Saleem et al. presented an end-to-end security-enabled intelligent remote *IoT* monitoring system focusing on secure data acquisition and transmission. The proposed architecture employs *AES* 256-bit encryption to protect sensor data at the device level before transmission over a cellular network to cloud storage. Encrypted data can be accessed through a mobile application that performs decryption based on authorized user input. The system is designed to mitigate common security threats, including brute-force, man-in-the-middle, phishing, spoofing, and *denial-of-service (DoS)* attacks. Experimental validation using a real testbed demonstrates the reliability of the proposed system in terms of encryption delay and achievable data rates. However, the study primarily addresses secure data monitoring and transmission, and does not consider secure *OTA* firmware update mechanisms or their integration with automated firmware build and deployment pipelines.

In [31], Promila et al. proposed a *Trusted Platform Module (TPM)*-based secure boot mechanism for embedded systems to enhance hardware-level security. The proposed procedure enforces mandatory signature verification through the *TPM*, preventing the system from booting unless firmware integrity is successfully validated. This approach ensures that unauthorized code modifications are detected early in the boot process and that the system always starts in a well-defined and trusted state. The mechanism was evaluated on different embedded platforms to analyze boot time variations and the impact of enabling secure boot during system update procedures. However, the work primarily focuses on secure boot at system startup and does not address secure *OTA* firmware update mechanisms or their integration with automated firmware build and deployment pipelines.

Overall, existing firmware security frameworks primarily concentrate on strengthening device-level trust anchors, secure boot processes, hardware-based key management, or encrypted communication channels. These approaches significantly enhance system integrity and resistance against physical and network-based attacks. However, they often treat firmware security as an isolated runtime or boot-time protection mechanism rather than as a continuous lifecycle process spanning firmware development, automated building, secure signing, controlled release, and *OTA* deployment. In particular, limited attention has been given to integrating secure firmware verification and authentication mechanisms within automated *CI/CD* pipelines to ensure reproducible and verifiable firmware releases. Consequently, a unified end-to-end framework that bridges firmware-level security primitives with automated deployment workflows remains insufficiently addressed in the current literature.

## 3. Proposed System

In this section, we present a *over-the-air (OTA)* firmware update system implemented using a *CI/CD*-based approach.

### 3.1. System Architecture

The proposed system consists of hardware and software components that support the firmware *over-the-air* update process. An update process begins when a developer modifies the source code or fixes bugs for an *IoT* device and subsequently commits and pushes the code to a repository (GitHub) integrated with a *hosted runner*.

In this paper, the *CI/CD* process is executed by a *CI/CD runner*, which is a dedicated execution environment responsible for performing automated tasks defined in the pipeline, including firmware compilation, cryptographic hashing and signing, and artifact packaging. The *hosted runner* operates on a centralized server and is automatically triggered upon code changes in the repository. After the build process is completed, which requires approximately four minutes, the firmware undergoes hashing, signing, and packaging stages. The resulting firmware package is then delivered to the *SEMAR IoT* application server, which publishes an update notification via the *MQTT* protocol secured with *TLS*.

In the proposed architecture, an edge computing layer is introduced to bridge the *ESP32*-based microcontrollers and the Internet. The edge computing layer plays a crucial role in improving scalability and reducing direct communication overhead between a large number of *IoT* devices and the *SEMAR IoT* application server. By aggregating *OTA* update requests, the edge layer significantly reduces the number of direct requests sent to the application server when the number of deployed devices increases.

In this paper, the edge computing layer is implemented using a Raspberry Pi 4. Upon receiving the update notification from the *SEMAR IoT* application server, the edge computing node downloads the firmware package through an *API Gateway*. The *API Gateway* provides a *Representational State Transfer (REST)* interface over the *HTTPS* protocol and acts as a single entry point to the application server, thereby enhancing security through service isolation and controlled access.

**Figure 1:**
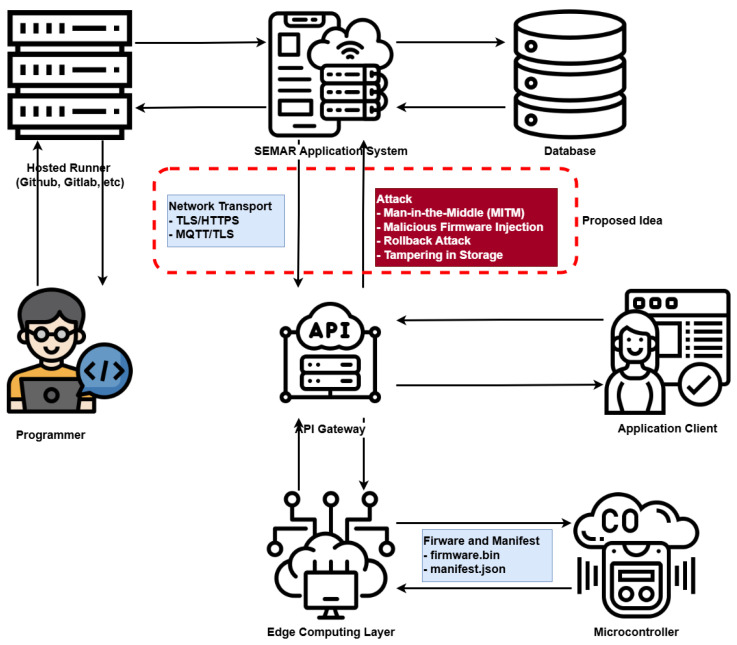
Architecture of the *CI/CD*-enabled *OTA* update system.

After the firmware package is successfully downloaded, the edge computing node forwards the update notification to the microcontroller. The *ESP32* device then retrieves the firmware package from the edge node via the *HTTPS* protocol. Once the download process is completed, the firmware is updated on the microcontroller. All *OTA* update activities, from firmware retrieval by the edge computing layer to firmware installation on the *ESP32*, can be monitored through the *application client*, which is implemented as a web-based monitoring interface. Figure 1 illustrates the complete architecture of the proposed firmware *over-the-air* update process.

As illustrated in Figure 1, the proposed *OTA* firmware update workflow follows a layered and sequential process. The workflow starts from the developer side and proceeds through the *CI/CD runner* for automated build and signing, followed by firmware distribution via the *SEMAR IoT* application server. The edge computing layer acts as an intermediate aggregation and security layer before the firmware is securely delivered to the *ESP32*-based devices. This figure provides an end-to-end overview of the interactions among system components and clarifies the role of each layer in ensuring secure and scalable *OTA* updates.

### 3.2. Threat Model

This subsection explicitly defines the trust assumptions and adversarial capabilities considered in the proposed *OTA* architecture. The trusted components include the secure bootloader and firmware verification logic embedded in the *IoT* device, the public key provisioned on the device for signature verification, the *CI/CD runner* responsible for automated firmware compilation and signing, the secure secret storage mechanism protecting the signing private key within the *CI/CD* environment, and the official *SEMAR IoT* application server infrastructure. These components are assumed to operate correctly and are not compromised.

The adversary is modeled as a remote attacker with full control over the communication network. The attacker may intercept, modify, replay, or redirect network traffic, including performing *MITM* attacks, DNS manipulation, firmware replay attempts, version rollback attempts, and injection of modified firmware binaries. However, the attacker is assumed to have no physical access to the device hardware and cannot extract cryptographic material from secure storage within the device or the *CI/CD* secret management system.

Compromise of the signing private key or the *CI/CD* execution environment is considered outside the immediate threat model but represents a residual supply-chain risk. If the signing infrastructure is compromised, a malicious firmware image could be legitimately signed and distributed. To mitigate such risks in practical deployments, industry best practices such as hardware-backed key storage (e.g., HSM or KMS), protected repository branches, reproducible builds, provenance attestations, and audit logging mechanisms are recommended.

Under these assumptions, the proposed *OTA* mechanism focuses on preventing unauthorized firmware distribution, rollback and replay attacks, network-level manipulation, and integrity violations during firmware delivery while ensuring that no unverified firmware is activated on the device.

### 3.3. Firmware Update Workflow

The firmware update workflow provides a more detailed overview of the *over-the-air* firmware update mechanism, from the initial development stage to the installation process on an *IoT* device. The workflow begins when a developer commits and publishes a source code change. Subsequently, the *hosted runner* builds the project to generate a firmware binary file. The generated firmware binary is digitally signed using a cryptographic signing algorithm with a private key securely stored in the repository platform (e.g., GitHub). The private key is managed using a secret mechanism, ensuring that it is not publicly accessible and can only be updated by replacing the key. In addition to the signing process, a hashing procedure is performed to ensure the firmware integrity and to generate versioning information. The firmware binary and signing information, or manifest, are then packaged into an *OTA* archive to reduce file size and facilitate efficient distribution. The *hosted runner* uploads the firmware package to the *SEMAR* server via the *HTTPS* protocol using a *REST API*. Upon receiving the firmware package, the *SEMAR* server publishes a firmware update notification using the *MQTT* protocol secured with *TLS*.

The integrated workflow between the developer’s code modification process and the automated firmware packaging and publication performed by the *hosted runner* constitutes the *CI/CD* process. This process is fully automated and executed without human intervention, thereby minimizing the risk of errors during firmware updates.

**Figure 2:**
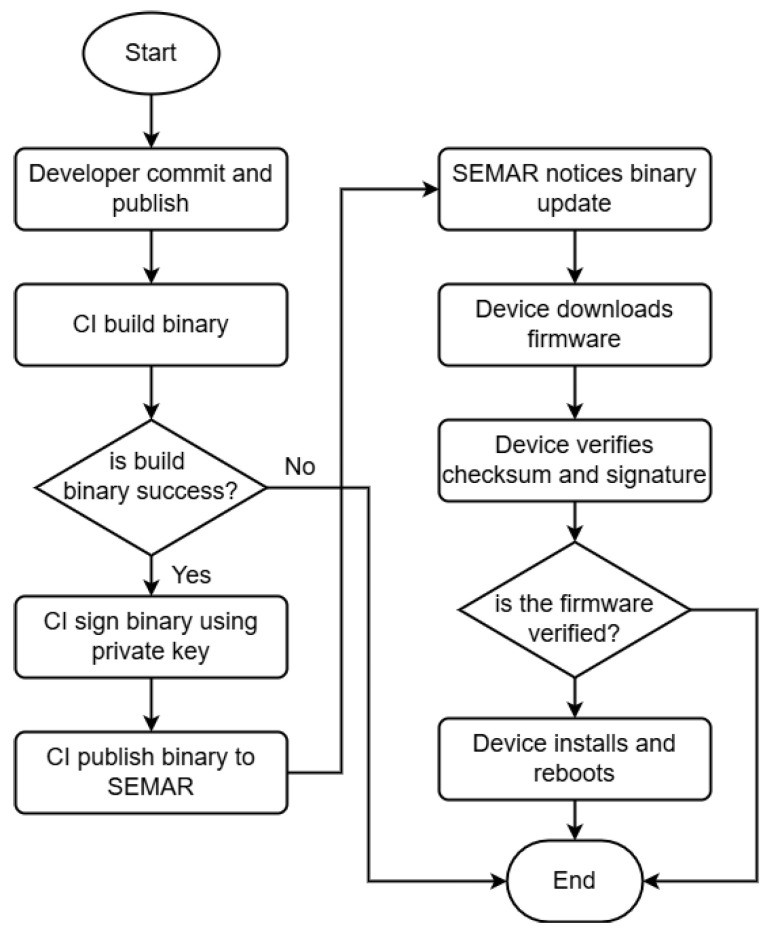
Detailed *OTA* firmware update workflow process.

Once the *IoT* device receives the update notification, it downloads the firmware package via the *HTTPS* protocol. Due to the limited storage capacity of an *IoT* device, the firmware package is downloaded using a chunk- or buffer-based mechanism. The downloaded firmware package is temporarily stored in *Serial Peripheral Interface Flash File System (SPIFFS)* storage in the archive form before being extracted and written to the firmware partition. During the extraction process, the firmware version, hash, and digital signature information are retrieved and verified. These verification steps ensure that the firmware version is the latest, the hash matches the downloaded file, and the digital signature is valid. The signature verification is performed using the public key stored on the *IoT* device. If any verification step fails, the *over-the-air* firmware update process is terminated. Otherwise, the *IoT* device installs the new firmware and reboots to operate using the updated firmware. Figure 2 illustrates the complete firmware update workflow in detail.

### 3.4. Security Mechanisms

The security mechanism is applied across both the communication protocols and the firmware generation process. The communication layer employs https and MQTT protocols, both secured using TLS. The https protocol is used when the *runner* distributes the firmware package to the *SEMAR* server and when the *IoT* device downloads the firmware package from the server. To establish secure communication with the *SEMAR* server, the *IoT* device requires a digital certificate, which is generated during the TLS configuration process on the physical server. Securing the communication protocols aims to prevent data interceptions and *MITM* attacks during firmware package distribution.

The next security layer is applied during the firmware package generation process on the *runner*. The firmware build process includes signing, hashing, and versioning stages. The signing process employs the *ED25519* algorithm, which is well known for its lightweight and fast performance on *IoT* devices. The private key used for signing is provisioned to the *CI/CD* environment through the repository secret management mechanism, which restricts direct exposure in source code and limits access to the automated signing process. The resulting digital signature is verified by the *IoT* device to confirm that the firmware was generated by the trusted *runner*. The verification process is performed using the corresponding public key stored on the *IoT* device. Hashing is performed to ensure firmware integrity by detecting any modification to the firmware file after it has been downloaded. Finally, the versioning mechanism combines timestamp information and an incrementing build iteration to ensure that outdated firmware versions are not installed on *IoT* devices.

### 3.5. Key and Certificate Lifecycle Management

Secure lifecycle management of cryptographic keys and certificates is a critical component of the proposed *OTA* architecture.

The firmware signing private key is generated offline and manually provisioned into the *CI/CD* environment as an encrypted repository secret (e.g., GitHub Actions secret). The private key is never stored in the source code repository and is only accessible during the automated firmware signing process executed by the hosted *runner*. Any key replacement requires manual regeneration and secure re-provisioning within the *CI/CD* secret storage.

For device-to-server communication, Transport Layer Security (TLS) is implemented using certificate authority (CA) validation. On ESP32 platforms, the esp_crt_bundle_attach mechanism is used to embed a trusted root CA bundle within the firmware image. This approach allows the device to validate server certificates without requiring firmware updates when server certificates are renewed, provided that they chain to a trusted root CA.

The firmware authenticity verification process relies on a public key embedded in the device firmware at provisioning time. This public key acts as a trust anchor for signature verification of *OTA* firmware packages. In the event of key rotation or compromise, a transitional firmware update signed with the existing trusted key can be used to deploy a new public key. After successful installation, subsequent firmware releases may use the updated signing key.

Currently, automated certificate revocation and dynamic key rotation mechanisms are not implemented and remain as future enhancements. Nevertheless, the proposed architecture supports controlled key replacement through staged firmware updates.

### 3.6. Integration with CI/CD Pipelines

**Figure 3:**
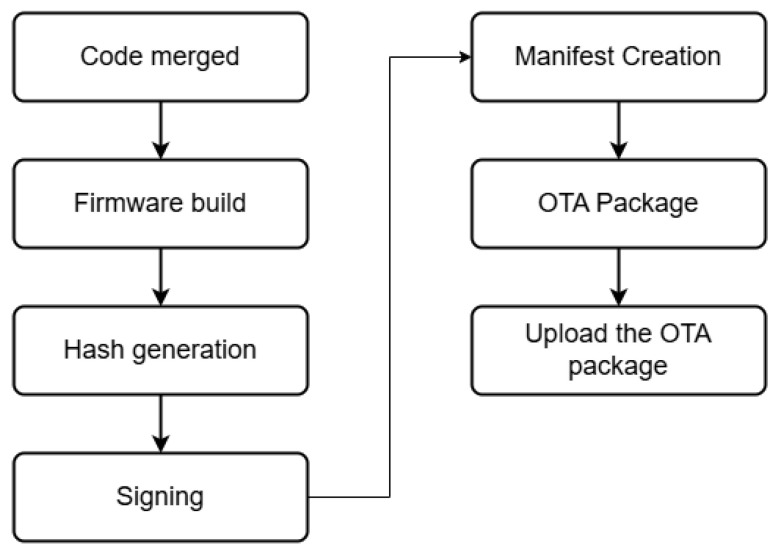
Pipeline stages.

The *CI/CD* pipeline consists of a series of automated processes in software development used to build, test, and consistently distribute changes in source code. The primary objective of *CI/CD* is to ensure that these processes are executed repeatedly and consistently without human intervention, thereby minimizing potential errors. In the context of *IoT* systems, *CI/CD* is employed to automate firmware compilation, configuration validation, and *over-the-air* firmware distribution in a controlled and secure manner. The *CI/CD* process is executed on a *runner*, which acts as an execution environment provided by the *CI/CD* platform (e.g., GitHub Actions). The general *CI/CD* workflow, illustrated in Figure 3, consists of sequential stages including source triggering, firmware build, validation, artifact generation, digital signing, and secure deployment.

In the proposed implementation, the *CI/CD* pipeline is automatically triggered when a developer pushes or commits code to the designated branch in the version control repository. The *hosted runner* is activated based on this event and executes a predefined workflow described in a *YAML* configuration file. This workflow defines the sequential stages of the automated build process, including environment initialization, dependency installation (e.g., ESP-IDF setup), firmware compilation, artifact generation, digital signing procedures, and packaging of the *OTA* firmware image.

All stages are executed autonomously by the *runner* without manual intervention. During the digital signing stage, cryptographic keys and sensitive credentials are accessed through encrypted platform-level secrets configured within the *CI/CD environment*. These secrets are injected as protected environment variables at runtime and are not stored in plaintext within the source repository or exposed in build logs. After successful signing and verification, the generated firmware artifact is uploaded to the *OTA server (SEMAR)* for controlled distribution to *IoT* devices.

### 3.7. Integration with SEMAR IoT Server

The *SEMAR IoT application server*, which was previously developed by our research group receives requests, once the firmware packaging process has been completed by the *runner*. Communication is performed using the https protocol via a REST API, through which the firmware package is uploaded and stored on *SEMAR*. The stored firmware package is subsequently downloaded by an *IoT* device. Prior to the download process, *SEMAR* publishes a notification indicating the availability of a new firmware version using the MQTT protocol secured with TLS. *IoT* devices that subscribe to this notification then download the firmware package from the server using the https protocol.

## 4. Evaluation and Results

In this section, the results demonstrate the proposed *OTA* firmware update system and present the results of the conducted experiments.

### 4.1. Experimental Setup

For the experiments, the *SEMAR IoT application server* was deployed on a cloud service. Table 1 presents the detailed hardware and system configuration of the deployed server, including its computing resources and operating environment used to support the *OTA* update evaluation.

As the *IoT* device used in the *over-the-air* firmware update process, an *ESP32* microcontroller was selected. Table 2 summarizes its key technical specifications, including processor architecture, memory capacity, wireless connectivity features, and hardware cryptographic support relevant to the *OTA* implementation.

The *ESP32* firmware employs a dual *OTA* partition scheme to enable seamless firmware updates while maintaining system reliability. Table 3 presents the complete partition layout configuration used in this paper. The dual *OTA* approach allows the device to switch between two firmware partitions (*app0* and *app1*), ensuring that a valid firmware is always available even if an update fails. The *SPIFFS* partition is utilized for temporary storage of the downloaded firmware package before extraction and installation.

To execute the *CI/CD* pipeline, *GitHub Actions* was utilized, employing a *runner* with a *Linux (x64) operating system, 4 vCPUs, 16 GB of RAM, and 16 GB of SSD storage*. The selected runner configuration corresponds to a *standard GitHub-hosted runner* available in the free-tier service. This environment represents a typical cloud-based *CI/CD* execution setting rather than a specialized or high-performance infrastructure. The computational resources (4 vCPUs and 16 GB RAM) were sufficient to handle firmware compilation, dependency setup (ESP-IDF), artifact generation, and digital signing processes without requiring hardware acceleration or dedicated build servers. Therefore, the chosen configuration reflects a realistic and reproducible *DevOps* deployment scenario for embedded firmware automation.

### 4.2. Security Testing Results

Several security testing scenarios were conducted to evaluate the robustness of the proposed *OTA* firmware update mechanism. The tests focused on firmware integrity, version rollback prevention, source authentication, and resistance against *MITM* attacks.

#### 4.2.1. Firmware Integrity Verification

*Firmware integrity* testing was performed to evaluate resistance against firmware tampering during the *OTA* update process. The firmware successfully built and stored on *SEMAR* was downloaded and manually modified at the binary level by changing a single byte without re-signing the firmware. The modified firmware was then repackaged and redeployed to the *OTA* server, and an update notification was triggered. After the *ESP32* device downloaded the firmware, a hash verification process was executed and compared with the hash value stored in the firmware manifest. Since the binary had been altered, the computed hash differed from the expected value, causing the *OTA* update process to be rejected. This result demonstrates that firmware tampering or binary modification is detected during the verification stage, preventing unauthorized firmware installation under the defined threat model.

#### 4.2.2. Version Rollback Protection

To evaluate protection against *version rollback* attacks, *OTA* updates were performed using different firmware versions. A previous firmware version stored on *SEMAR* was retained while the device was updated to the latest firmware version. Subsequently, the firmware on the *OTA* server was replaced with the older version, and an update notification was triggered. During the update process, the *ESP32* device detected a version mismatch and rejected the installation. The firmware versioning scheme follows the format [hex_commit]-[timestamp]-[build_number], where the hex commit represents an 8-digit commit identifier from the source repository, the timestamp indicates the build time generated by the *CI runner*, and the build number increments with each build. For example, 84788c4-20251219T0210-build97 represents the 97th build generated on 19 December 2025, at 02:10. This unique versioning mechanism ensures that outdated firmware cannot be reinstalled.

#### 4.2.3. Unauthorized Firmware Source Detection

*Unauthorized source* testing was conducted by modifying the *OTA* server address to a server using an untrusted TLS certificate. When the *ESP32* device attempted to download the firmware, the *HTTPS* connection failed during the *TLS* handshake process due to certificate verification errors. As a result, the *OTA* update process was aborted, demonstrating that firmware updates can only be performed from authorized and trusted sources.

#### 4.2.4. Resistance Against MITM Attacks

*Resistance against MITM attacks* was evaluated by simulating intercepted *OTA* communication within the same local network using a *Raspberry Pi*. *Address Resolution Protocol (ARP) spoofing* was employed to position the Raspberry Pi between the ESP32 device and the *OTA* server during the firmware update process. When *ARP* spoofing was active, the *ESP32* device failed to establish MQTT over TLS and HTTPS connections, indicating that secure communication channels could not be established under interception conditions, resulting in termination of the *OTA* process. Consequently, the *OTA* update process was aborted, demonstrating effective protection against *MITM*-based attacks.

Overall, the security testing results demonstrate that the proposed *OTA* firmware update mechanism effectively mitigates common security threats, including firmware tampering, version rollback, unauthorized firmware sources, and *MITM* attacks, under the defined threat model in Section 3.2. Table 4 details the evaluated attack scenarios and their corresponding outcomes, confirming that the implemented validation layers function correctly under both network-level and firmware-level adversarial conditions.

#### 4.2.5. Extended Threat Model and Failure Behavior

To strengthen the security evaluation under more realistic adversarial conditions, additional threat scenarios were examined, including *replay attempts, network disruption during firmware download, DNS manipulation, and manifest tampering*. Table 5 summarizes these scenarios and their outcomes, demonstrating that the proposed mechanism maintains integrity and prevents unauthorized updates even under extended attack conditions.

In the *replay scenario*, a previously valid and signed firmware package with an older version number was resent to the device after a newer version had already been installed. The device rejected the update based on monotonic version validation before installation, preventing downgrade and replay-based attacks.

To evaluate resilience against *partial-download corruption*, Wi-Fi connectivity was intentionally disabled during firmware streaming. The TLS session was aborted, resulting in transport read failures and termination of the *OTA* process. The update was safely aborted without partition switching, and the device continued operating using the previously verified firmware.

For *DNS manipulation testing*, the *OTA* server domain was redirected to a malicious endpoint. TLS certificate validation failed due to root CA mismatch via esp_crt_bundle_attach, and the *OTA* process was aborted before any firmware download occurred.

In the *manifest tampering scenario*, version metadata was altered without re-signing the firmware package. Signature verification failed during validation, causing immediate rejection of the update. Since version information is protected by digital signature, bypass attempts were unsuccessful.

Across all evaluated scenarios, the *OTA* mechanism consistently enforced layered validation (TLS authentication, manifest integrity verification, signature validation, and version monotonicity checks). Any failure resulted in safe termination prior to firmware activation, ensuring that no partial or unverified firmware was installed.

### 4.3. Performance Analysis Results

To evaluate the performance of the proposal during the *over-the-air (OTA)* firmware update process for *IoT* devices, a comparative analysis was conducted between an unsecured communication protocol (*HTTP*) and a secure communication protocol (*HTTPS*). The firmware size after the build process was approximately *1.1 MB*, while the compressed update package size was reduced to approximately *635 kB*. The *OTA* firmware update process on the *IoT* device consists of several stages, ranging from *download_manifest* to *ota_finalize*. For each stage, the execution duration, heap memory usage, CPU utilization, and stack usage were measured.

#### 4.3.1. OTA Stage Execution Time Analysis

**Figure 4:**
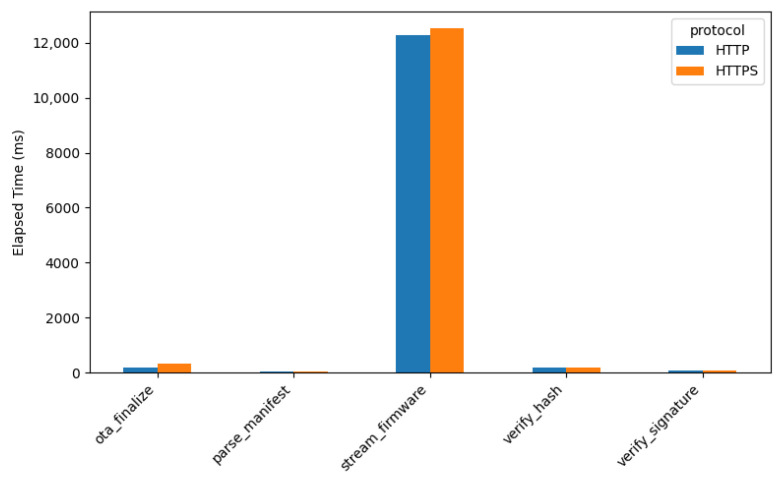
Average *OTA* stage duration (HTTP vs. HTTPS).

Figure 4 presents a comparison of the execution time required for each firmware update stage using HTTP and HTTPS.

The results indicate that the most significant difference occurs during the *stream_firmware* stage, as it involves streaming the firmware directly from *SEMAR*. In contrast, during subsequent stages, the firmware has already been stored locally on the *IoT* device, resulting in *negligible performance differences* between HTTP and HTTPS. Quantitatively, the *stream_firmware* stage exhibits an average execution time increase of approximately *2%* when HTTPS is used. Additionally, the *ota_finalize*, *parse_manifest*, *verify_hash*, and *verify_signature* stages also experience slight increases, although these remain statistically insignificant. This additional latency is primarily caused by the *TLS handshake process* and *encrypted data transfer*, which introduce communication overhead in HTTPS.

#### 4.3.2. Heap Memory Utilization

**Figure 5:**
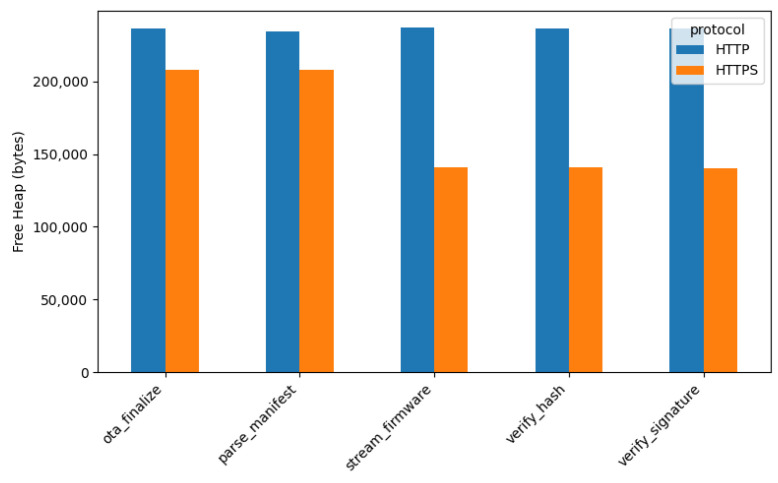
Minimum free heap memory during *OTA* (HTTP vs. HTTPS).

*Free heap memory* utilization was evaluated to investigate the impact of applying secure communication protocols during the *OTA* firmware update process. As illustrated in Figure 5, the HTTPS protocol consistently exhibits *lower free heap memory* compared to HTTP across all *OTA* stages. This reduction is primarily caused by additional memory allocations required by HTTPS, including *TLS RX/TX buffers*, *certificate chains*, and *TLS context structures*, which increase heap consumption during the update process.

#### 4.3.3. CPU Utilization

**Figure 6:**
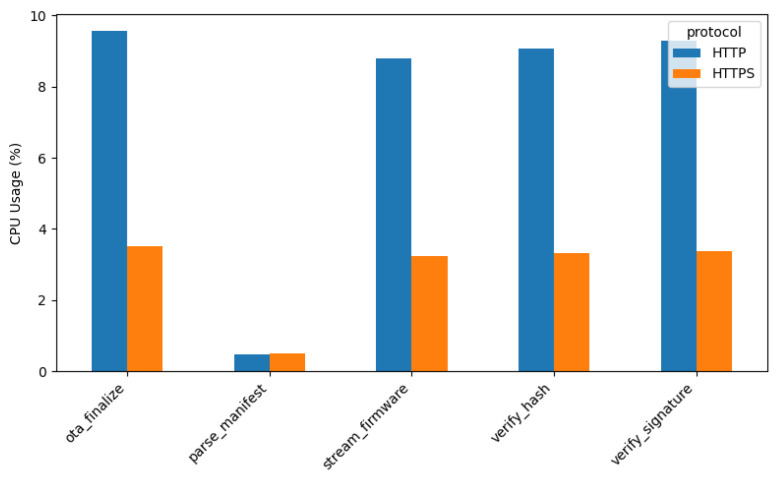
CPU utilization during *OTA* (HTTP vs. HTTPS).

Figure 6 presents the CPU utilization observed during the *OTA* process for both HTTP and HTTPS protocols. Overall, CPU usage remains relatively stable at approximately *5–6%* across most *OTA* stages. The only exception is the *parse_manifest* stage, where CPU activity is minimal since this phase primarily performs lightweight operations such as version comparison and hash validation prior to initiating the firmware update.

Although HTTPS requires additional cryptographic processing, the ESP32’s *hardware encryption accelerator* offloads TLS-related computations from the main CPU. As a result, CPU utilization under HTTPS remains comparable to that of HTTP. These findings indicate that while HTTPS introduces additional memory overhead due to TLS context management, it does not impose a significant computational burden on the processor, thereby enabling secure *OTA* updates without noticeable CPU performance degradation.

#### 4.3.4. Stack Memory Usage

**Figure 7:**
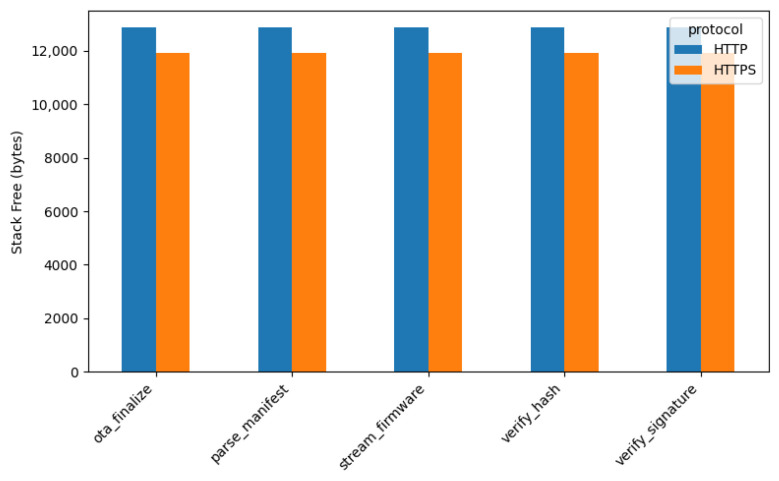
Free stack during *OTA* (HTTP vs. HTTPS).

The final performance parameter evaluated was *free stack usage* during the *OTA* firmware update process. Consistent with the previous metrics, the experimental results demonstrate that HTTP requires *higher stack memory consumption* compared to HTTPS, which can be attributed to the ESP32’s hardware cryptographic accelerator capabilities. When using the HTTPS protocol, the device exhibits approximately *7% lower stack usage* compared to HTTP. This reduced stack consumption is achieved despite HTTPS requiring additional processing for *TLS handshake procedures*, *cryptographic temporary buffers*, *certificate parsing*, and *record processing*. The ESP32’s hardware acceleration offloads these cryptographic operations, resulting in more efficient stack utilization. Figure 7 compares the free stack usage for each firmware update stage under both communication protocols.

### 4.4. Benchmarking Results

To provide a more in-depth analysis of the *over-the-air (OTA)* firmware update process, this paper compares cryptographically equivalent digital signature algorithms, namely *ECDSA-256* and *ECDSA-384*, and evaluates the *OTA* performance across multiple firmware sizes. The firmware size is incrementally increased from 1 MB to 5 MB, with the initial firmware size being approximately 1 MB. The evaluated performance metrics include update duration, CPU utilization, stack usage, and heap memory consumption.

#### 4.4.1. Impact of Firmware Size on OTA Performance

The *OTA* stages focus on *stream_firmware*, *verify_hash*, and *verify_signature*, as these stages are the most affected by the choice of digital signature algorithm and firmware size during the *OTA* update process. These stages directly involve cryptographic operations and data processing that scale with the firmware size.

**Figure 8:**
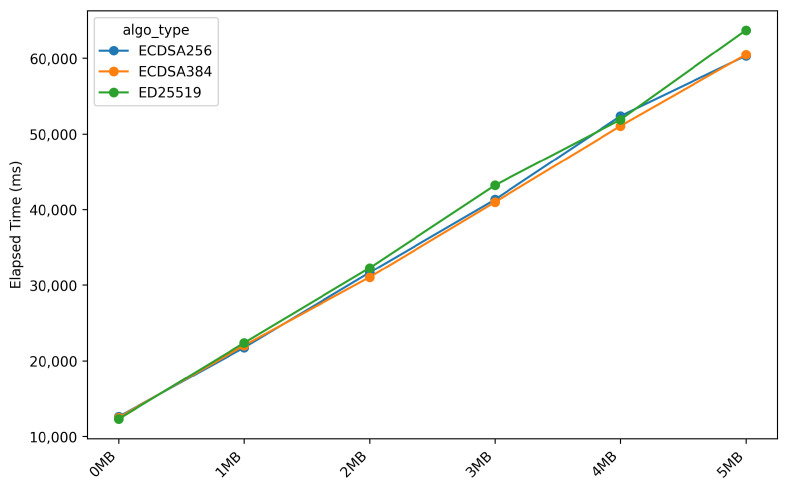
Elapsed time during stream firmware stage across different firmware sizes.

**Figure 9:**
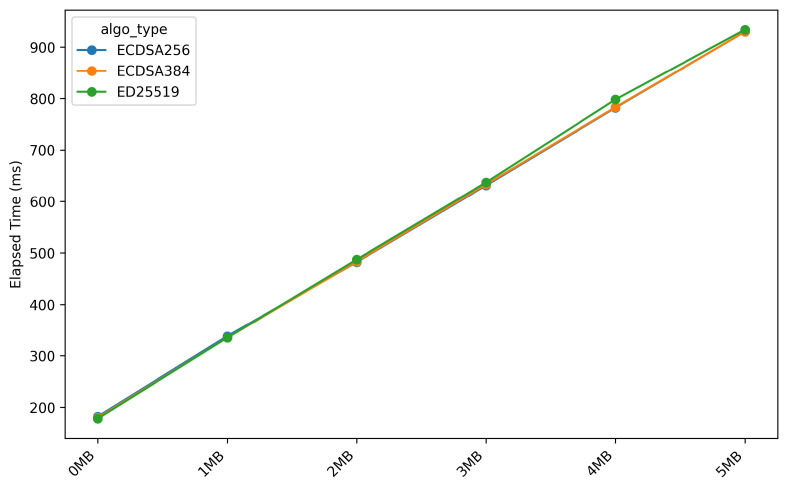
Elapsed time during verify hash stage across different firmware sizes.

Figure 8 and Figure 9 respectively illustrate the duration required for the *OTA* update process during the *stream_firmware* and *verify_hash* stages.

Figure 8 and Figure 9 show a strong correlation between firmware size and execution time for the *stream_firmware* and *verify_hash* stages. This relationship is confirmed by correlation analysis, yielding a statistically significant *p-value of 0.001* and a *coefficient of determination ($R^{2}$)* of 0.99, indicating an almost perfect model fit. For the *stream_firmware* stage, each additional 1 MB of firmware increases the execution time by approximately 10 s, while the *verify_hash* stage requires an additional 150 milliseconds per megabyte. This behaviour is expected, as both stages are dominated by I/O-bound operations and hash-dependent processing, causing execution time to scale proportionally with firmware size.

#### 4.4.2. Signature Verification Performance Comparison

**Figure 10:**
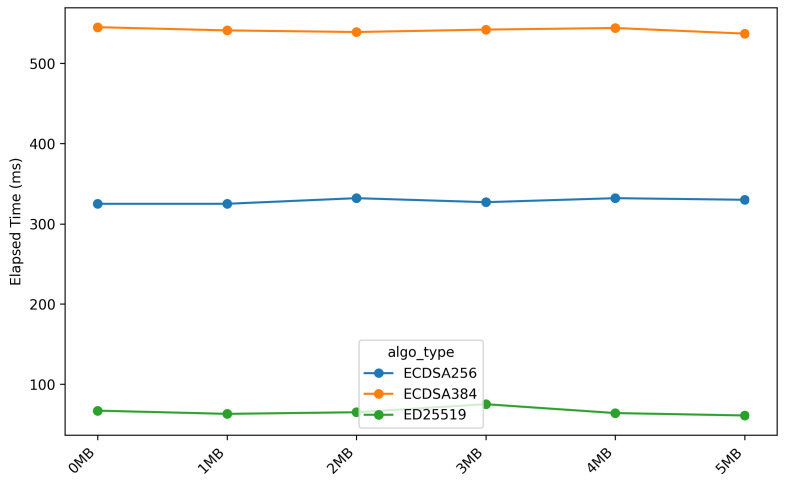
Elapsed time during verify signature stage across different firmware sizes.

Figure 10 illustrates the execution time during the *verify_signature* stage across different firmware sizes. In contrast, the *verify_signature* stage is not affected by firmware size, as indicated by a near-zero *correlation coefficient* of −0.0003 and a consistent execution time of approximately 310–315 milliseconds across all firmware sizes. This observation can be explained by the fact that signature verification operates on a fixed-size hash output rather than the raw firmware data, making it computation-bound rather than I/O-bound.

Furthermore, for the *stream_firmware* and *verify_hash* stages, the performance differences across the evaluated digital signature algorithms remain minimal, with variations of approximately 4% and nearly identical execution times. This result confirms that these stages are independent of the chosen signature algorithm. In contrast, significant performance differences are observed in the *verify_signature* stage. The *ED25519* algorithm requires approximately 65.83 milliseconds, whereas *ECDSA-256* requires 328.50 milliseconds, corresponding to a 399% increase compared to *ED25519*. *ECDSA-384* exhibits the highest latency at approximately 541.33 milliseconds, representing a 722% increase relative to *ED25519*. These results demonstrate that *ED25519* is approximately 5–8 times faster than *ECDSA-256* and *ECDSA-384*, primarily due to the more efficient *Edwards curve arithmetic* employed by *ED25519* compared to *ECDSA*, which relies on larger key sizes (256-bit versus 384-bit).

#### 4.4.3. Memory Consumption Analysis

**Figure 11:**
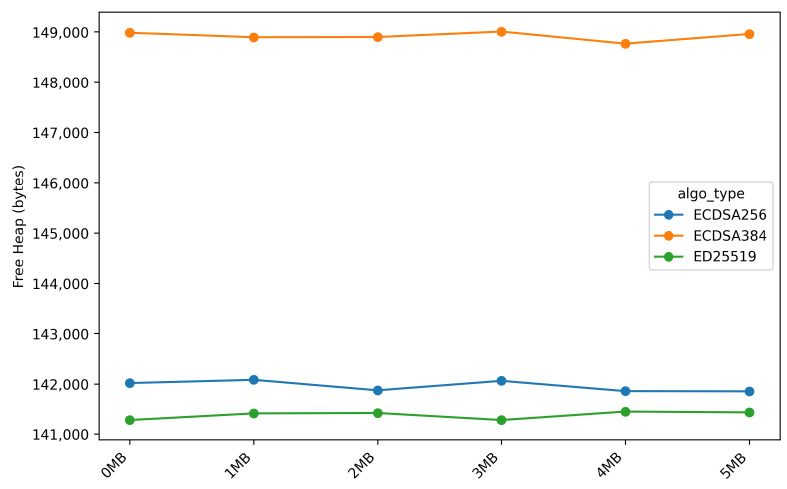
Free heap memory during stream_firmware stage across different firmware sizes.

**Figure 12:**
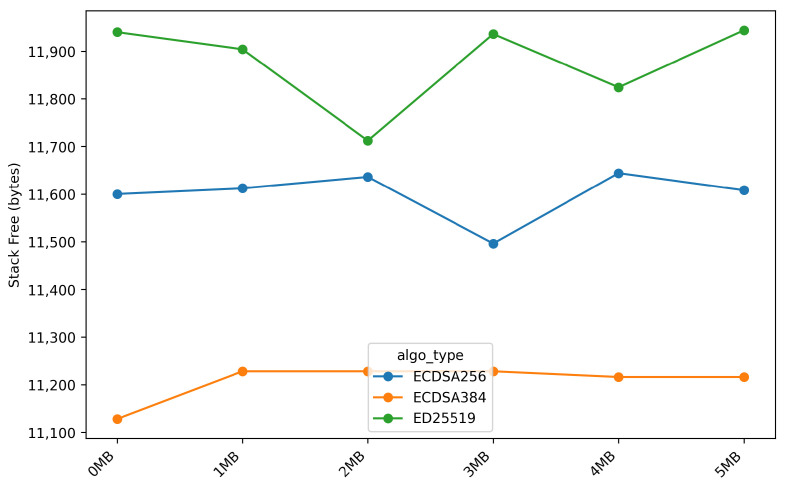
Free stack memory during stream_firmware stage across different firmware sizes.

Figure 11 and Figure 12 present a comparison of free heap and free stack memory usage during the *OTA* firmware update process, focusing on the *stream_firmware* stage, as the remaining *OTA* stages exhibit identical memory usage patterns. This behavior is attributed to the *ESP32* platform, which provides *hardware acceleration* support for *multi-precision integer arithmetic* in several digital signature algorithms.

The memory analysis shows that the available free heap memory is approximately 148.9 KB for ECDSA-384, 142 KB for ECDSA-256, and 141.4 KB for ED25519. The 5.1% heap memory advantage of ECDSA-384 over ED25519 indicates that software-based cryptographic implementations incur higher memory overhead for operations such as point multiplication tables, temporary workspaces in finite-field arithmetic, and Edwards curve conversion buffers.

A similar trend is observed for stack memory usage. ECDSA-384 requires approximately 11.2 KB of stack memory, ECDSA-256 requires 11.6 KB, and ED25519 requires 11.9 KB. The 5.6% stack advantage of ECDSA-384 over ED25519 suggests slightly shallower call chains in the software implementation of ECDSA-384. Nevertheless, all evaluated algorithms maintain safe stack utilization levels, remaining well below critical thresholds (less than 50% stack consumption). Overall, the memory evaluation confirms that all evaluated signature algorithms are well suited for *OTA* deployment on resource-constrained IoT devices.

#### 4.4.4. Cross-Platform Validation on Different MCU Families

To address concerns regarding platform-specific conclusions, additional experiments were conducted on multiple MCU families, namely *ESP8266MOD*, *ESP32-WROOM*, and *ESP32-S3*. The objective is to validate whether the performance observations of ED25519 and ECDSA-256 remain consistent across different hardware architectures and software stacks.

It should be noted that the ESP32 series (*ESP32-WROOM* and *ESP32-S3*) were implemented using the official ESP-IDF framework, whereas *ESP8266MOD* was implemented using the Arduino core. This choice is necessary because ESP-IDF officially supports only the *ESP32* series, while *ESP8266MOD* is not supported under ESP-IDF. Therefore, each MCU was evaluated using its officially supported and widely adopted development framework to ensure realistic and ecologically valid deployment conditions.

Table 6 summarizes the average verification time and free heap memory during the *verify_signature* stage.

The results indicate that performance differences between *ED25519 and ECDSA-256* vary across MCU architectures and software stacks. On *ESP8266MOD* (*Arduino core*), both algorithms exhibit comparable verification time, with *ECDSA-256* slightly faster, suggesting similar software-based computational overhead. On *ESP32-WROOM* (*ESP-IDF*), *ECDSA-256* significantly outperforms *ED25519*, likely due to hardware acceleration support for multi-precision arithmetic. Conversely, on *ESP32-S3* (*ESP-IDF*), *ED25519* achieves lower verification time than *ECDSA-256*, indicating architectural optimization differences among *ESP32* variants. Free heap variations remain minor across platforms, confirming that both algorithms are feasible for *OTA* verification on resource-constrained devices. These results demonstrate that cryptographic performance is influenced by the interaction between algorithm design and hardware capabilities rather than being strictly platform-specific.

#### 4.4.5. Energy and Power Consumption Analysis

**Figure 13:**
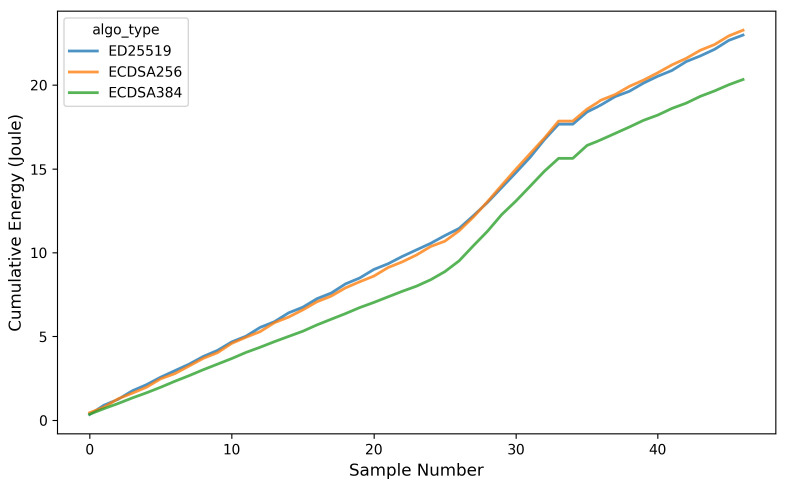
Energy consumption during *OTA* firmware update across different signature algorithms.

**Figure 14:**
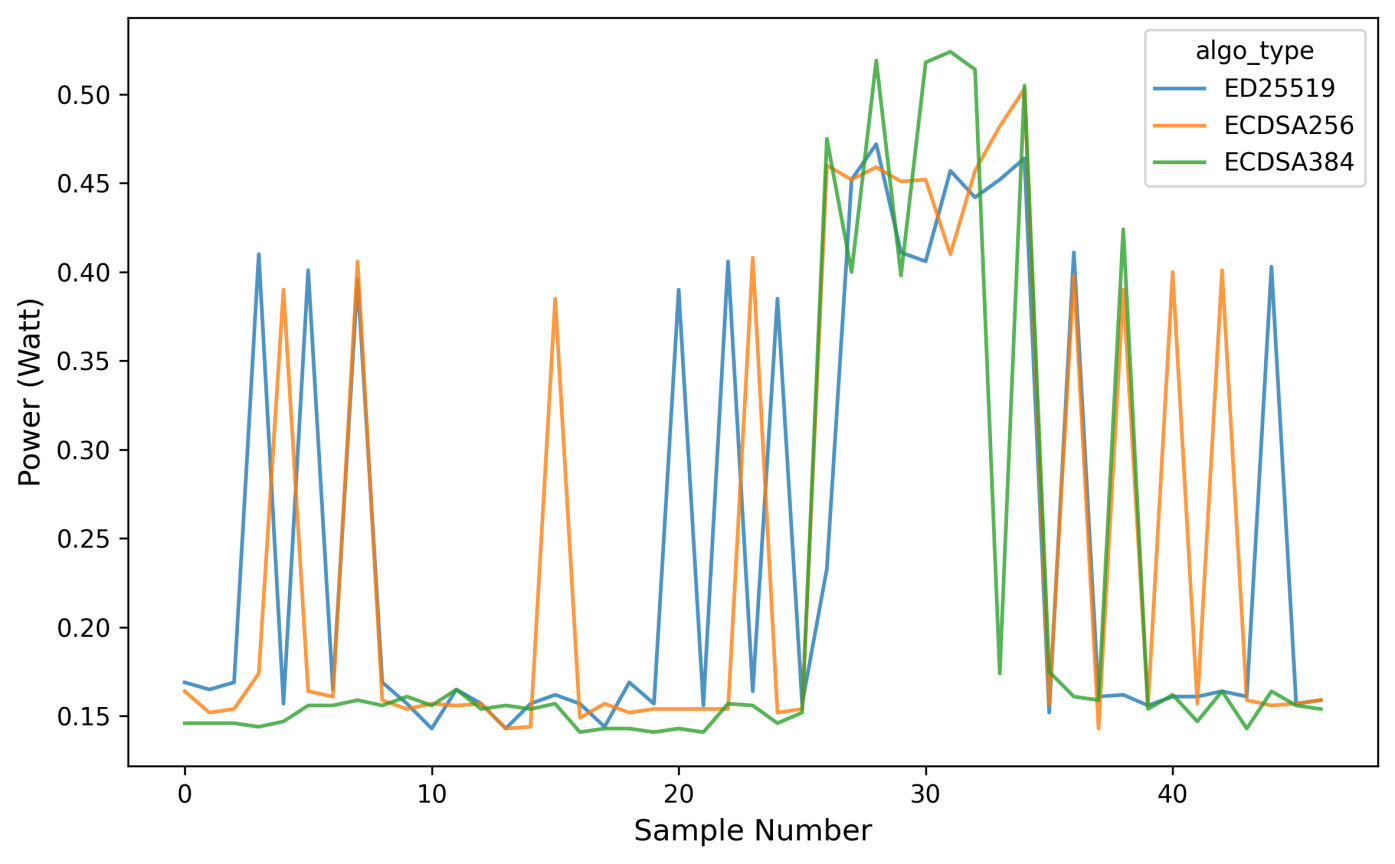
Power consumption during *OTA* firmware update across different signature algorithms.

Furthermore, the analysis of the energy consumption (Joule) and the power usage (Watt) during the *OTA* firmware update process for each signature algorithm is presented in Figure 13 and Figure 14. Measurements were performed by sampling power data every 100 ms and transmitting the collected data to the server every 2 s to minimize request overhead. As shown in Figure 14, the *OTA* update process for all three signature algorithms exhibits a clear increase in power consumption compared to the idle state, particularly between sample numbers 25 and 35. This interval corresponds to the active *OTA* phase, including firmware download, signature verification, and flash writing operations, which collectively impose higher computational and I/O loads on the ESP32.

The results indicate that ECDSA-P384 achieves approximately 11–13% lower energy consumption compared to ED25519. Although ECDSA-P384 exhibits longer execution times and larger key sizes, the ESP32 benefits from hardware acceleration for *Multi-Precision Integer (MPI)* operations, which significantly reduces CPU activity. As a result, the instantaneous power consumption during signature verification is lower, leading to reduced overall energy usage.

### 4.5. CI/CD-Integrated vs. Standalone OTA Deployment Comparison

To clarify the contribution of *CI/CD* integration, this paper compares the proposed system with a standalone secure *OTA* deployment. In the standalone configuration, firmware is built locally using *ESP-IDF*, followed by *(1) source checkout, (2) firmware compilation, (3) hash generation, (4) digital signature creation, (5) manual upload to the* OTA *server, and (6) manual update triggering*. In contrast, the proposed configuration integrates *GitHub Actions* to automatically perform compilation, signing, and artifact distribution upon a *single commit event*, with the private signing key securely stored as an environment secret.

Table 7 summarizes the operational and security differences between the two approaches. Both configurations provide HTTPS-based transport security and on-device signature verification, ensuring firmware authenticity and integrity. However, the *CI/CD-integrated* approach reduces manual intervention (six steps to one step), eliminates direct private key handling, and binds each firmware artifact to a specific commit and build identifier, improving traceability and supply-chain control.

Although CI-based builds require approximately four minutes due to *runner provisioning and toolchain initialization*, compared to less than one minute for local builds, this overhead is negligible in typical *OTA* release cycles. The primary benefit of *CI/CD* integration lies not in reducing build time but in strengthening operational security and end-to-end deployment governance.

### 4.6. Discussion

This paper proposes an *end-to-end over-the-air (OTA)* firmware update mechanism for *IoT* devices that integrates a *CI/CD* pipeline to automatically distribute firmware packages without human intervention. The proposed mechanism aims to minimize operational errors while maintaining a high level of security against common *OTA* threats, including *version rollback attacks, unauthorized firmware sources, and MITM* attacks. Lightweight digital signature algorithms are employed to ensure firmware authenticity and integrity while preserving computational efficiency.

As summarized in Table 4, the experimental results demonstrate that all evaluated attack scenarios failed to disrupt or compromise the *OTA* update process, indicating the effectiveness of the proposed security mechanisms. Furthermore, the use of the ED25519 signature algorithm for firmware verification shows superior performance in terms of execution time, achieving verification speeds approximately 5–8 times faster than ECDSA-256 and ECDSA-384. This performance advantage is mainly attributed to the efficiency of *Edwards curve arithmetic*, which enables faster signature verification compared to ECDSA-based schemes.

From a memory efficiency perspective, ED25519 requires slightly higher heap and stack memory usage compared to ECDSA-based algorithms. This behaviour can be explained by the presence of a hardware accelerator for *multi-precision integer arithmetic* on the ESP32 platform, which optimizes certain ECDSA operations and reduces their memory overhead. These results highlight a trade-off between execution speed and memory efficiency when selecting digital signature algorithms for resource-constrained *IoT* devices.

Compared to related works, many existing firmware update mechanisms still rely on manual user interventions, such as physically uploading firmware images and manifest files to trigger the update process [5]. Other approaches focus on decentralized infrastructures, such as *IOTA* and the *Inter Planetary File System (IPFS)*, to enhance firmware authenticity and integrity in smart home IoT environments [32]. Additionally, some studies address specific security threats by employing lightweight cryptographic schemes tailored for constrained devices [13].

Table 8 provides a qualitative comparison between these representative approaches and the proposed *CI/CD-integrated OTA* framework. As shown in the table, most existing solutions primarily focus on secure firmware transmission or signature verification, but do not integrate automated *CI/CD* workflows, enforce strict rollback protection, or evaluate resilience under network interruption conditions. In contrast, the proposed system unifies deployment automation, end-to-end cryptographic protection, and failure resilience within a single operational pipeline.

Despite its advantages, this work has several limitations. The firmware image size remains relatively large (approximately 1 MB), which may increase update duration or lead to update failures under unstable network conditions. Moreover, the current implementation stores firmware packages on the *SEMAR IoT application server*, requiring reliable network connectivity during the *OTA* update process. These factors may affect scalability in deployments with intermittent or constrained network access.

One potential approach to mitigate the firmware size limitation is the integration of delta (differential) update mechanisms, where only binary differences between firmware versions are transmitted instead of the full image. In principle, delta artifact generation can be incorporated into the automated *CI/CD* pipeline during the build stage, allowing differential packages to be generated and signed prior to deployment. However, integrating delta updates within the proposed end-to-end security framework introduces additional challenges. These include maintaining end-to-end signature integrity across base and patch images, ensuring strict version synchronization between the installed firmware and the corresponding delta package, and preserving rollback protection guarantees. Furthermore, secure verification must ensure that patch reconstruction does not introduce integrity inconsistencies. Therefore, while delta updates represent a promising extension for bandwidth-constrained environments, careful redesign of the signing scope and version control mechanisms would be required.

Although ED25519 demonstrates superior execution time performance, ECDSA-based algorithms may offer better memory efficiency on platforms equipped with *cryptographic hardware accelerators*. Therefore, the selection of digital signature algorithms should consider both computational performance and memory constraints based on the target deployment environment. In addition, secure communication is enforced through HTTPS, which introduces only negligible overhead due to the ESP32’s hardware encryption acceleration, making it suitable for secure *OTA* firmware updates in *IoT* systems.

## 5. Conclusions

This paper presented a secure *end-to-end over-the-air (OTA)* firmware update system for *IoT* devices integrated with a *CI/CD* software distribution model. The proposed approach effectively mitigates critical security threats, including *version rollback attacks*, *unauthorized firmware sources*, and *MITM attacks*, by combining lightweight digital signature algorithms with secure communication protocols. Experimental results demonstrate that *ED25519* significantly outperforms *ECDSA*-based schemes in firmware signature verification, achieving approximately 5–8× faster execution time, while the use of *HTTPS* introduces only marginal overhead during the firmware streaming stage. Furthermore, CPU utilization remains consistently low across all *OTA* processing stages, indicating that secure firmware updates can be efficiently performed on resource-constrained *IoT* devices. As future work, the *OTA* update pipeline can be extended by offloading firmware compilation and packaging to a gateway device, enabling more robust update execution under unstable network conditions while preserving end-to-end security guarantees.

## Figures and Tables

**Table 1:** Node specifications.

Specification	Details
CPU	1 Core
RAM	1 GB
Storage	25 GB
OS	Ubuntu 24.04.3 LTS

**Table 2:** IoT device specifications.

Specification	Details
Microcontroller	ESP32 (Xtensa LX7 architecture)
CPU	Dual-core 32-bit processor
Clock frequency	Up to 240 MHz
ROM	448 KB
SRAM	520 KB
External Flash	16 MB SPI flash
Wireless Connectivity	2.4 GHz Wi-Fi (802.11 b/g/n), Bluetooth v4.2 BR/EDR + BLE
Cryptographic Hardware	AES, SHA, RSA, ECC accelerator
Hardware RNG	True random number generator (TRNG)
Secure Boot Support	Yes
Flash Encryption	Yes
Operating Voltage	3.0 V–3.6 V

**Table 3:** ESP32 partition layout for *OTA* firmware updates.

Name	Type	SubType	Offset	Size	Flags
nvs	data	nvs	0 × 9000	0 × 5000	-
otadata	data	ota	0 × e000	0 × 2000	-
phy_init	data	phy	0 × 10,000	0 × 1000	-
app0	app	ota_0	0 × 20,000	0 × 770,000	-
app1	app	ota_1	0 × 790,000	0 × 770,000	-
spiffs	data	spiffs	0 × F00000	0 × 100,000	-

**Table 4:** Summary of security testing scenarios and results.

Threat Scenario	Test Method	Result
Firmware tampering	Binary modification (hex editor)	Update rejected
Version rollback	Older firmware version	Update blocked
Unauthorized source	Invalid server certificate	Connection refused
MITM attack	HTTPS certificate mismatch	OTA aborted

**Table 5:** Extended security testing results.

Threat	Test Method	Result
Replay	Older signed firmware resent	Rejected (version check)
Partial download	Wi-Fi disabled during OTA	Aborted safely
DNS poisoning	Domain redirected	TLS handshake failed
Manifest tampering	Metadata modified without re-signing	Signature failed

**Table 6:** Average verification time and free heap across different MCU platforms.

Device	Framework	Algorithm	Time (ms)	Free Heap (Bytes)
ESP8266MOD	Arduino Core	ED25519	507.3	23,664
		ECDSA-256	484.3	23,592
ESP32-WROOM	ESP-IDF	ED25519	421.3	100,967
		ECDSA-256	58.0	98,579
ESP32-S3	ESP-IDF	ED25519	67.0	141,036
		ECDSA-256	325.0	142,020

**Table 7:** Comparison between standalone *OTA* and *CI/CD*-integrated *OTA* deployment.

Metric	Standalone OTA	CI/CD-Integrated OTA
HTTPS transport security	Yes	Yes
On-device signature verification	Yes	Yes
Manual deployment steps	6	1
Manual private key handling	Required	Not required
Artifact traceability	Limited	Commit-bound
Supply-chain integrity	Partial	End-to-end
Average build time	<1 min	∼4 min

**Table 8:** Comparison with related standalone *OTA* approaches.

Aspect	[5] Bhargava et al.	[13] Park et al.	Proposed
CI/CD integration	No	No	Yes
Deployment automation	Manual	Manual	Automated
End-to-end security	Partial	Partial	Yes
Rollback protection	No	Limited	Yes
Network resilience	No	No	Yes
Cross-platform support	Single	Limited	Multiple

## Data Availability

Data are contained within the article.
